# Veterinary Medical Society of the Ontario Veterinary College

**Published:** 1897-12

**Authors:** C. W. Fisher

**Affiliations:** Secretary


Secretary.

### VETERINARY MEDICAL SOCIETY OF THE ONTARIO VETERINARY COLLEGE.

THE Society held its first meeting of this college session, October 13th. The Society was called to order by Prof. Sweetapple in a few well-chosen remarks, after which the Society proceeded to its annual election of officers, which resulted as follows: Secretary, C. W. Fisher; Assistant Secretary, J. S. Pollard; Treasurer, R. Macdonald; Librarian, W. L. Adams. The meeting then adjourned. Since then weekly meetings have been held, presided over by one of the members of the Faculty, and the following papers read by members of the senior class and duly discussed by all.

Essays: "Navicular Disease," S. S. Treadwell; "Chloral Hydrate," Alex. McGregor; "Arnica," R. Macdonald; "Mange in Dog," C. E. S. Brind; "Tuberculosis," W. H. Pethick; "Navicular Disease," A. E. Atwood; "Shoeing," E. R. Stockwell; "Contagious Diseases of Swine," A. C. Walker; "Examination for Soundness," G. W. Mackie; "Castration," J. E. Ellis.

Communications: "Parturient Paralysis," J. A. Raleigh; "Removal of Eyeball of Dog," Alex. McGregor; "Sunstroke," C. E. S. Brind; "Indigestion in Dog," I. W. Parks; "Acute Laminitis," E. B. Shaw; "Ascites in Dog," R. Macdonald; "Simple Ophthalmia," H. R. Clark; "Purpura Hemorrhagica," J. P. Stranghan; "Parturient Apoplexy," G. W. Mackie; "Inversion of Uterus," J. S. Pollard; "Lipoma of Eye," F. J. Neiman; "Thrombosis of Metacarpal Artery," A. E. Atwood; "Poison in Dog," J. D. Bell; "Choking," B. W. Powell; "Acute Indigestion," D. McKenzie; "Mammitis," W. H. Pethrick.

C. W. FISHER,
Secretary.

